# *Plasmodium falciparum* Histidine-Rich Protein II Compromises Brain Endothelial Barriers and May Promote Cerebral Malaria Pathogenesis

**DOI:** 10.1128/mBio.00617-16

**Published:** 2016-06-07

**Authors:** Priya Pal, Brian P. Daniels, Anna Oskman, Michael S. Diamond, Robyn S. Klein, Daniel E. Goldberg

**Affiliations:** a Department of Molecular Microbiology, Washington University School of Medicine, St. Louis, Missouri, USA; b Department of Pathology and Immunology, Washington University School of Medicine, St. Louis, Missouri, USA; c Division of Infectious Diseases, Department of Medicine, Washington University School of Medicine, St. Louis, Missouri, USA; d Department of Neuroscience, Washington University School of Medicine, St. Louis, Missouri, USA

## Abstract

Cerebral malaria (CM) is a disease of the vascular endothelium caused by *Plasmodium falciparum*. It is characterized by parasite sequestration, inflammatory cytokine production, and vascular leakage. A distinguishing feature of *P. falciparum* infection is parasite production and secretion of histidine-rich protein II (HRPII). Plasma HRPII is a diagnostic and prognostic marker for falciparum malaria. We demonstrate that disruption of a human cerebral microvascular endothelial barrier by *P. falciparum*-infected erythrocytes depends on expression of HRPII. Purified recombinant or native HRPII can recapitulate these effects. HRPII action occurs via activation of the inflammasome, resulting in decreased integrity of tight junctions and increased endothelial permeability. We propose that HRPII is a virulence factor that may contribute to cerebral malaria by compromising endothelial barrier integrity within the central nervous system.

## IMPORTANCE

Cerebral malaria is a devastating disease. Patients have high levels of the protein HRPII in their blood. We have found that endothelial cell barriers become leaky when treated with concentrations of HRPII similar to those found in patients. This result suggests that HRPII may be important in cerebral malaria. Our finding that HRPII functions by causing inflammation suggests points of intervention for therapy or vaccination against this disease.

## INTRODUCTION

The clinical presentation of malaria ranges from a febrile illness (uncomplicated malaria) to life-threatening disease, including severe anemia, respiratory distress, and cerebral malaria (CM) (1). *Plasmodium falciparum* contributes the greatest morbidity and mortality and is the species that causes CM. CM results in about 300,000 deaths annually, has a 20% case fatality rate despite treatment (2–5), and 25% of survivors have long-term neurological sequelae, including cognitive impairment (6). CM patients present acutely with decreased sensorium, progressing to coma. This neurological syndrome is characterized by sequestration of infected red blood cells (RBCs) in cerebrovascular beds, vascular occlusion, inflammation, perivascular edema, and brain swelling (7–9). Brain swelling and perivascular edema are strongly associated with death in CM (9). These manifestations are due in part to breakdown of the blood-brain barrier (BBB). The BBB regulates access of solutes and cells to the central nervous system and includes a complex network of endothelial intercellular junctional proteins (basement membranes), with ensheathment by pericytes, and astrocyte end-feet. Disruption of this network results in BBB compromise and has been linked to a variety of disease states (11).

Histidine-rich protein II (HRPII) is a unique protein produced exclusively by *P. falciparum*; 37% of its amino acid sequence is histidine, and repeats of histidine plus alanine cover 85% of its sequence. HRPII is exported by the parasite into the RBC cytosol (12). As parasites rupture from the host cell, RBC cytosolic components, including HRPII, are released into the bloodstream. In plasma, HRPII can reach 100 µg/ml. Since its discovery in 1986 (13), many functions have been ascribed to it, including hemozoin crystallization, actin formation, T cell suppression, glycosaminoglycan binding, and procoagulation (14–17).

HRPII has been used as a biomarker for *P. falciparum* infection and forms the basis of many current rapid diagnostic tests (18, 19). On postmortem analyses, HRPII has been observed to line the endothelial walls of blood vessels (20). Several correlative studies showed an association between plasma HRPII levels and disease severity or development of CM (18, 21–25). Natural populations of HRPII-deficient *Plasmodium falciparum* parasites exist (26–28), though these tend to be in areas of low CM incidence.

Due to the established correlation between HRPII levels and cerebral malaria (18, 24, 25), we questioned whether HRPII contributes directly to disease pathogenesis. We provide evidence that HRPII is a *P. falciparum* virulence factor that triggers the inflammasome in vascular endothelial cells. HRPII binding to brain endothelial cells results in rearrangement of tight junction proteins and a compromised blood-brain barrier (BBB). We propose that HRPII contributes to the pathogenesis of cerebral malaria.

## RESULTS

### HRPII compromises endothelial barrier integrity.

**FIG 1:**
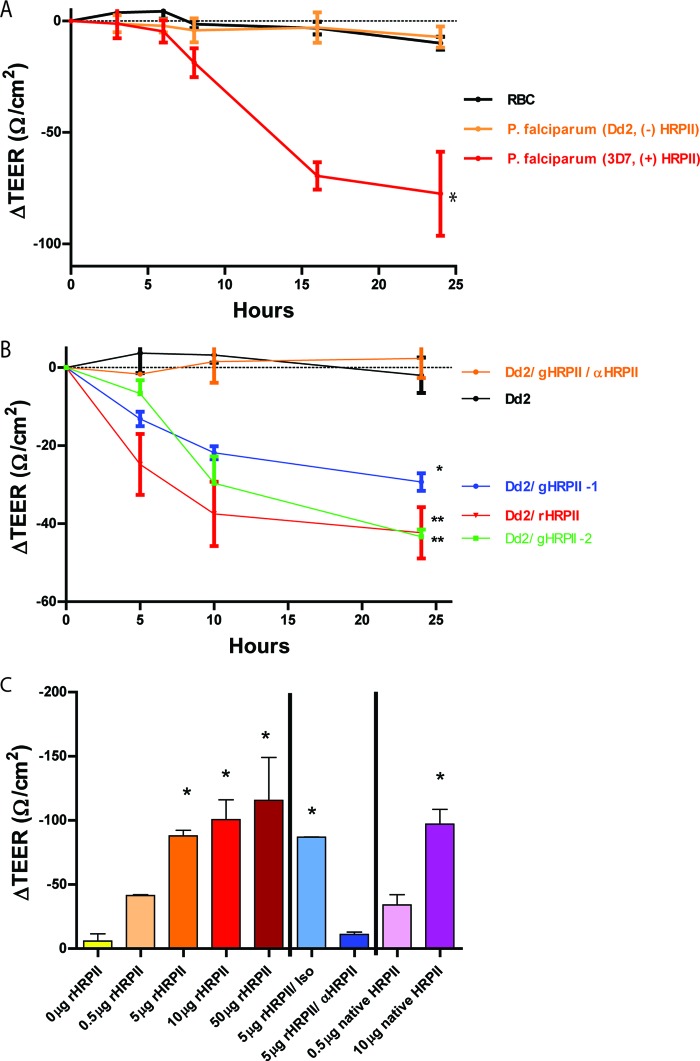
HRPII is both necessary and sufficient to compromise the integrity of an *in vitro* endothelial barrier. TEER was measured across an hCMEC/D3 monolayer over time, and components for assessment were added to the upper chamber (1-ml volume). (A) 10^8^ uninfected RBCs or *Plasmodium falciparum* strain Dd2 (which does not produce HRPII) or strain 3D7 (which produces HRPII) cells were added to the model BBB. All values are relative to resistance measurements at time zero. Data are mean values ± standard errors of the means (SEM) of results from 2 biological replicates performed in triplicate. Dd2 was significantly different from 3D7 by one-way analysis of variance (ANOVA) (×, *P* < 0.05). (B) Addition of 10^8^ Dd2 parasites, parasites engineered to produce HRPII (Dd2/gHRPII-1 and Dd2/gHRPII-2), Dd2 parasites with 10 µg of added recombinant HRPII (Dd2/rHRPII), or Dd2/gHRPII-1 parasites in the presence of specific antibody (Dd2/gHRPII/anti-HRPII [αHRPII]). Data are mean values ± SEM of results from 6 replicates from three independent experiments. *, *P* < 0.0001; **, *P* < 0.009 (by one-way ANOVA for differences from Dd2-treated cells). (C) Addition of recombinant purified HRPII (rHRPII) or of HRPII purified from 3D7 parasites (native HRPII) or in combination with monoclonal anti-HRPII antibody (αHRPII) or isotype control (Iso). Data are mean values ± SEM of results from a 24-h time point for 4 replicates (most conditions), 6 replicates (antibodies), and 8 replicates (0 and 5 µg rHRPII), pooled from five independent experiments. *, *P* < 0.0001 (by one-way ANOVA for differences from 0 µg).

*P. falciparum* parasites as well as soluble parasite components have been shown to compromise the integrity of an *in vitro* BBB (29). We assessed the consequence of HRPII exposure in an *in vitro* BBB model that uses a previously established human cerebral microvascular endothelial cell line (hCMEC/D3) shown to behave like primary cells in their response to barrier perturbation (30). hCMEC/D3 monolayers display apicobasal polarity; the upper chamber of this cellular model represents the luminal face of a blood vessel (31). *P. falciparum* clone 3D7-parasitized erythrocytes were added to the upper chamber, and transendothelial electrical resistance (TEER) was measured across the endothelial barrier. These parasites induced a time-dependent decrease in resistance (Fig. 1A). In contrast, clone Dd2, which contains a deletion of the HRPII gene, caused minimal change in barrier integrity. Dd2 parasites were transfected to generate transgenic parasites that ectopically express HRPII. Integration of the gene for HRPII was confirmed by PCR, and isolated clones demonstrated an ability to produce HRPII by Western blotting (see Fig. S1 in the supplemental material). Two clones expressing HRPII from independent transfections compromised barrier integrity (Fig. 1B). Addition of a neutralizing anti-HRPII monoclonal antibody to the upper chamber confirmed the specific effect of HRPII, as it abolished the barrier compromise observed using the transfected parasites. Addition of recombinant, soluble HRPII to wells containing wild-type Dd2 parasites also resulted in barrier compromise. These experiments demonstrate that HRPII is required for parasites to disrupt endothelial barrier integrity *in vitro*. Purified HRPII alone (recombinant or isolated from *P. falciparum* 3D7 parasites) similarly disturbed barrier integrity in a dose-dependent manner, at the concentrations that are seen in the blood of patients with cerebral malaria (Fig. 1C). HRPII-mediated barrier compromise took several hours to develop and was maximal by 10 to 12 h, consistent with a requirement for new protein synthesis (see Fig. S2A). Disruption of the barrier was specific, as antibody blockade of HRPII abolished the effect. Equimolar concentrations of l-histidine, poly l-histidine, or peptides for the two main repeats in HRPII did not recapitulate the effect seen with HRPII (see Fig. S2B).

### HRPII induces redistribution of tight junction and adherens junction proteins.

**FIG 2:**
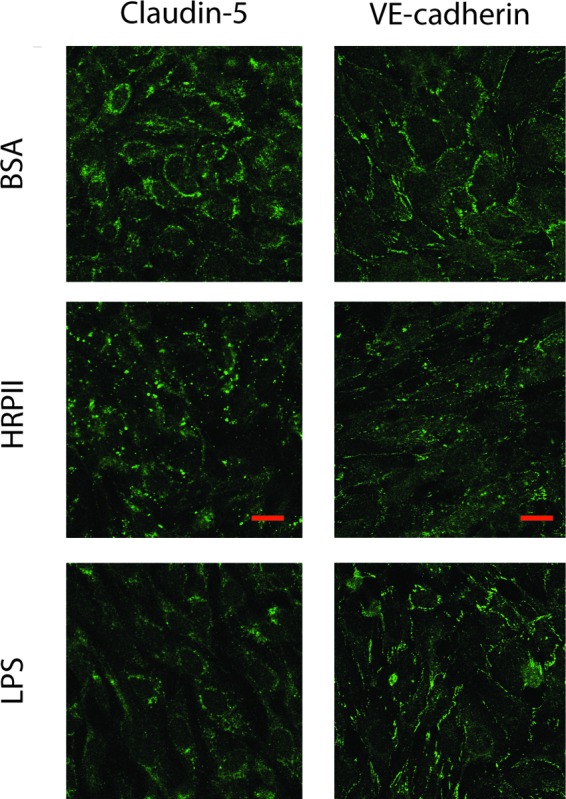
HRPII exposure to human cerebral microvascular endothelial cells results in redistribution of junctional proteins. hCMEC/D3 monolayer cultures were incubated for 24 h with 25 µg of BSA or HRPII or with 3 µg of LPS and were stained for tight junction protein claudin-5 and the adherens junction protein VE-cadherin. Representative images of 4 replicates from 2 independent experiments are presented. All images were taken using the same settings. Bar, 20 µm.

To assess whether HRPII compromises barrier integrity by altering the localization of BBB junctional proteins, we performed immunohistochemical analysis of hCMEC/D3 brain microvascular cells, staining for junctional proteins. This analysis revealed punctate redistribution of the tight junction protein claudin-5 and the adherens junction protein VE-cadherin (Fig. 2). The effects were similar to those observed with lipopolysaccharide (LPS), a Toll-like receptor 4 (TLR4) agonist that is known to disrupt the BBB.

### HRPII activates an innate immune response in endothelial cells.

**FIG 3:**
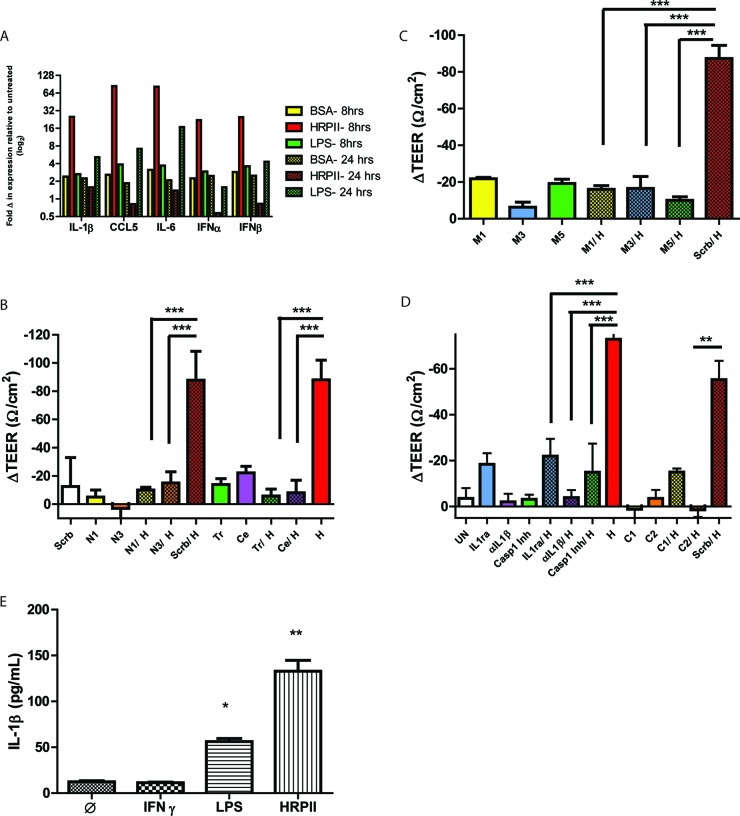
HRPII activates an inflammatory pathway in human cerebral microvascular endothelial cells. (A) qRT-PCR of chemokine/cytokine mRNA levels of hCMEC/D3 cells treated with 25 µg HRPII or BSA for 8 h and 24 h. (B) TEER measurements for *in vitro* hCMEC/D3 barriers transfected with shRNAs for NFκB (N1 and N3) or a scrambled control (Scrb) for 36 h or incubated with inhibitors for NFκB, celastrol (Ce), and triptolide (tr) for 2 h prior to addition of HRPII (H; 10 µg). Data are mean values ± SEM of results from 6 to 8 replicates pooled from three independent experiments. ***, *P* < 0.0001 (by one-way ANOVA). (C) TEER measurements for *in vitro* barriers transfected with shRNAs to MyD88 (M1 and M3 and M5) or a scrambled control (Scrb) for 36 h prior to addition of recombinant purified HRPII (10 µg). Data are mean ± SEM of results from 6 to 8 replicates pooled from 3 independent experiments. ***, *P* < 0.0001 (by one-way ANOVA). Results of assessment of knockdown levels are shown in Fig. S2 in the supplemental material. (D) TEER measurements for *in vitro* barriers transfected with shRNAs for caspase-1 (C1 and C2) or a scrambled control (Scrb) for 36 h or with IL-1Ra (500 ng), anti-IL-1β (αIL-1β) (25 ng), or the caspase-1 inhibitor YVAD-CMK (80 µM) (C1 Inh) for 1 h prior to treatment with recombinant purified HRPII (10 µg; H). Data are mean values ± SEM of results from 6 to 8 replicates pooled from four independent experiments. ***, *P* < 0.001 (by one-way ANOVA); **, *P* < 0.05 (by one-way ANOVA). (E) Quantitative ELISA for cleaved IL-1β from cell lysates. Cells were treated for 24 h with HRPII (10 µg), LPS (3 µg/ml), or IFN-γ (100 ng/ml) or left untreated. Data represent results from three biological replicates, each performed in triplicate. *, *P* = 0.0002; **, *P* = 0.0005 (compared to untreated control by unpaired *t* test).

The disruption of barrier integrity by HRPII suggested that endothelial cells recognize and respond to the protein. To investigate whether this response is mediated by a cell-intrinsic, host defense signaling response, we measured chemokine and cytokine transcripts by quantitative reverse transcription-PCR (qRT-PCR) analysis in hCMEC/D3 cells (32). We detected upregulation of transcripts within 8 h postexposure to recombinant HRPII, and the response was distinguished kinetically from that observed with LPS (Fig. 3A). The expression signature was suggestive of NFκB activation. To assess the involvement of NFκB directly, we blocked its action with short hairpin RNAs (shRNAs) and chemical inhibitors. Two different chemical inhibitors of NFκB subunit p65, triptolide (33) and celastrol (34), ablated the TEER changes induced by recombinant HRPII and resulted in elevated TEER and normalized barrier integrity (Fig. 3B). To corroborate the role of NFκB in HRPII-mediated effects on barrier integrity, we silenced expression of the p105 subunit of NFκB (see Fig. S3 in the supplemental material). Again, a decrease in TEER was prevented (Fig. 3B).

Many cellular host defense pathways activate NFκB signaling. Gene silencing of a common intracellular adaptor, MyD88 (see Fig. S3 in the supplemental material), performed using three different shRNAs, significantly reduced the HRPII-mediated drop in TEER (Fig. 3C). These data suggest that HRPII-mediated inflammation is NFκB and MyD88 dependent.

### HRPII activates the inflammasome.

MyD88 is an intracellular adaptor for several innate immune receptors, with some of these proteins using MyD88 as an exclusive intracellular adaptor: TLR1 and TLR2 (TLR1/2), TLR2/6, TLR5, TLR7, TLR9, interleukin-1 receptor (IL-1R), and IL-18R (10, 35). Silencing of TLR2, TLR5, and TLR9 (see Fig. S3 in the supplemental material) did not impact HRPII-mediated endothelial cell barrier disruption (see Fig. S4). TLR7 silencing could not be achieved and was not evaluated. We assessed the impact of IL-1R signaling by neutralizing its ligand IL-1β with a polyclonal antibody or by using a natural antagonist to the receptor, IL-1Ra. We demonstrated that HRPII-mediated change in TEER requires IL-1β activation and signaling (Fig. 3D). A requirement for caspase-1 was confirmed using two distinct shRNAs for caspase-1 as well as the caspase-1-specific inhibitor YVAD-CMK. Endothelial barriers treated with these reagents did not display a change in TEER in the presence of HRPII (Fig. 3D). HRPII treatment resulted in generation of cleaved IL-1β as determined by enzyme-linked immunosorbent assay (ELISA) (Fig. 3E). These data indicate that activation of the inflammasome is required for HRPII-mediated BBB disruption.

HRPII binds to and is internalized by hCMEC/D3 cells (see Fig. S5 in the supplemental material). Internalization could be required for HRPII action, but further support for this notion awaits identification of the endothelial receptor for HRPII.

### HRPII-induced cell death and loss of barrier integrity are kinetically distinct phenotypes.

**FIG 4:**
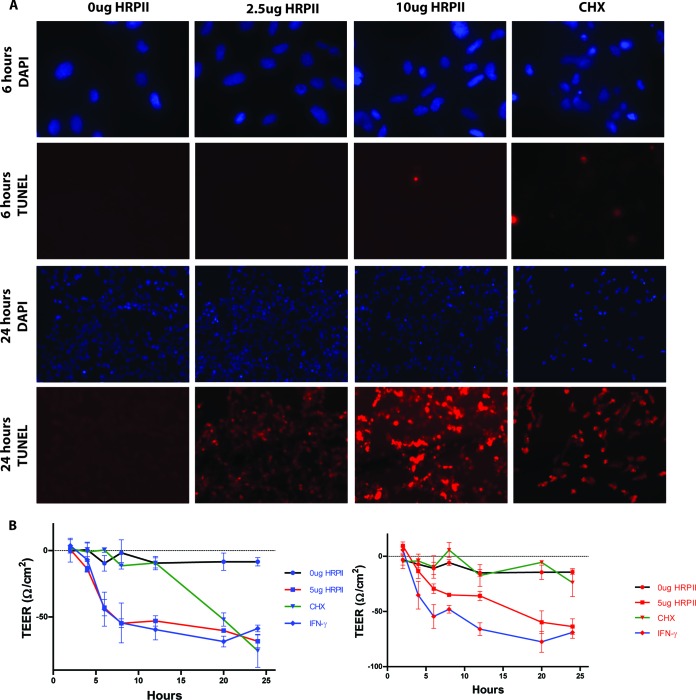
HRPII-mediated BBB compromise is independent of cell death. (A) HRPII results in late cell death, evidenced by nicked DNA in this TUNEL stain at 24 h postexposure to protein but not at 6 h. Cycloheximide (CHX) is a positive control for inducing cell death. (B) TEER measurements for *in vitro* model BBB left untreated or treated with IFN-γ (10 ng), HRPII, or CHX (10 ng/ml) in the absence (blue) or presence (red) of an apoptosis inhibitor (Z-VAD-FMK; 10 µg/ml). Data are means of results from 6 replicates ± SEM over three independent experiments.

Activation of the inflammasome can cause cell death. To determine whether endothelial cells lose viability in response to HRPII exposure, we monitored cell death at various time points. Cells undergoing programmed cell death display nicked DNA which can be visualized with a terminal deoxynucleotidyltransferase-mediated dUTP-biotin nick end labeling (TUNEL) stain. HRPII-treated cells showed no TUNEL staining at 6 h (when barrier disruption is evident), although nicking of cellular DNA was evident later, at 24 h postexposure (Fig. 4A). To rule out the possibility that HRPII-mediated loss of barrier integrity was a consequence of cell death, we reassessed TEER changes in the presence of a cell death inhibitor, Z-VAD-FMK. In this experiment, cycloheximide (CHX) served as a positive control for endothelial cell disruption via apoptosis. In the presence of Z-VAD-FMK, cell death (not shown) and barrier leakage (Fig. 4B) mediated by CHX was prevented. Gamma interferon (IFN-γ) causes a rearrangement of junctional proteins (36) and serves as a control for TEER changes that are not a consequence of cell death. IFN-γ compromised barrier integrity even in the presence of the apoptosis inhibitor. Similarly to the IFN-γ control, HRPII-mediated TEER changes were sustained when cells were treated with Z-VAD-FMK (Fig. 4B). Taken together, these data indicate that the loss of barrier integrity induced by HRPII is not a byproduct of cell death.

### HRPII treatment upregulates cytoadherence molecules on endothelial cells *in vitro.*

**FIG 5:**
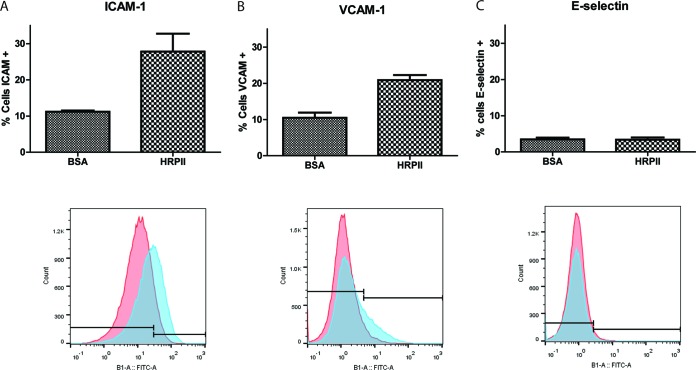
Human brain microvascular endothelial cells exposed to HRPII display an increase in levels of surface adhesion receptors. hCMEC/D3 cells were exposed to BSA or HRPII (10 µg) for 24 h. Surface expression of cell surface adhesion receptor was measured by flow cytometry. (A and B) Levels of ICAM-1 expression (A) and VCAM-1 expression (B) were significantly different as determined using a two-tailed *t* test (*P* = 0.02 for each). (C) E-selectin expression results were not significantly different between BSA treatment and HRPII treatment (*P* = 0.94). Representative flow cytometry histograms from three independent experiments in triplicate are shown below each bar graph.

Cerebral malaria is accompanied by upregulation of cytoadherence molecules on the vascular endothelium (37–39). We assessed the surface expression of several relevant adhesion receptors on human brain microvascular endothelial cells after treatment with HRPII. The percentage of cells expressing ICAM-1 and VCAM-1 was increased upon HRPII treatment (Fig. 5A and B). In contrast, E-selectin expression was not increased (Fig. 5C). ICAM-1 expression levels and binding by parasites are associated with severity of disease (40).

## DISCUSSION

BBB breakdown during *P. falciparum* infection is a significant feature of CM. The pathophysiology underlying this effect, however, is poorly understood. Our study has identified HRPII as a parasite virulence factor that activates the host innate immune system through an inflammasome-mediated pathway. This causes redistribution of endothelial junctional proteins and increased BBB permeability. A number of factors, including glycosylphosphatidylinositol (GPI), hemozoin, DNA, uric acid crystals, and microvesicles, have been proposed to activate pattern recognition receptors and/or inflammasomes. In many of these cases, however, the bloodstream concentration and therefore the physiological relevance are hard to ascertain (41). In contrast, HRPII plasma levels in malaria patients are easy to measure; incubation of endothelial cells or infusion of mice with physiologically relevant concentrations of HRPII led to the effects reported here.

*P. falciparum* parasites produce two highly homologous histidine-rich proteins: HRPII and HRPIII. Of the two, HRPII is produced abundantly, whereas HRPIII accumulates at much lower levels (42). The Dd2 *P. falciparum* parasite strain produces only HRPIII and has a deletion in HRPII. Using this background strain, we generated transgenic parasites that express HRPII. Whereas parental Dd2 parasites caused minimal change in TEER in an *in vitro* BBB model with human cells, the HRPII-expressing clones caused a substantial decrease in TEER. HRPII, the major histidine-rich protein, appears responsible for this action on the endothelium.

Intracellular and bloodstream functions for HRPII have been proposed, including digestive vacuole heme sequestration and procoagulant activity, respectively (14, 17). There are other proteins that can sequester heme even in the absence of HRPs (14, 43), and the physiological contribution of HRPII to the procoagulant state seen in falciparum malaria has not been established (17). Severe malaria and cerebral malaria are the states that have been associated with high HRPII levels clinically, and it is reasonable to propose that the most important role of this molecule is in endothelial inflammation. There are field isolates that lack HRPII (26–28), but it is not known whether patients infected with these strains have a milder course or diminished progression to CM.

Assessment of transcriptional responses to HRPII suggested activation of the NFκB pathway. Gene silencing and antagonist experiments supported the idea of a role for MyD88 and NFκB in mediating the HRPII effect. Triptolide decreases expression of NFκB/p65 and increases expression of the cytosolic inhibitor IκB-α (33). Celastrol decreases expression and translocation of NFκB/p65 to the nucleus and diminishes cleavage and activation of IκB-α (34). In the presence of these inhibitors, the drop in TEER induced by HRPII was lost. However, despite extensive analysis, we did not identify an upstream pathogen recognition receptor. Rather, additional studies suggested involvement of the IL-1 receptor, as we could block HRPII-mediated barrier disruption with IL-1RA, a natural antagonist, or with neutralizing antibody to IL-1β. IL-1β is a cytokine that is activated from its proform by caspase-1. Caspase-1 is auto-catalytically processed when molecules are brought into proximity by the inflammasome. Consistent with a key role for the inflammasome, the HRPII effect on TEER was abolished by silencing of caspase-1 or treatment with a caspase-1 inhibitor.

Inflammasome activation has been previously implicated in falciparum malaria infections. Opsonization of parasitized red blood cells as well as of pooled patient sera from *P. falciparum* infections was shown to activate the inflammasome in macrophages (44). IL-1β also has been detected in histopathological sections from patients who died of cerebral malaria (45, 46).

**FIG 6:**
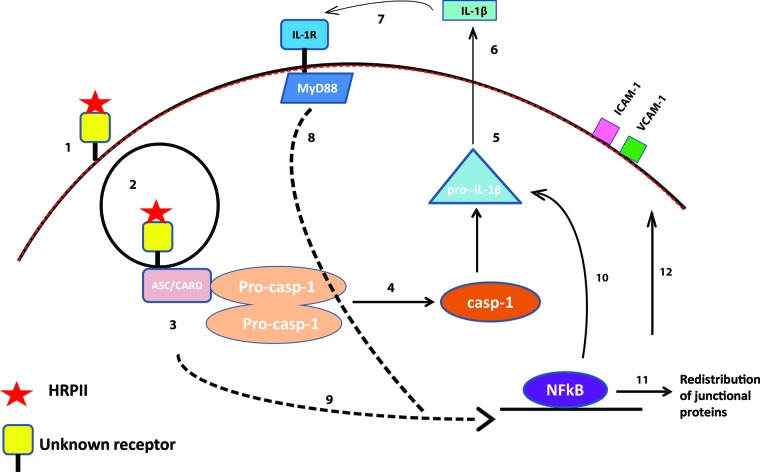
Model for HRPII recognition by human brain endothelial cells and the intracellular pathway that leads to BBB leakage. (1) HRPII binds to an as-yet-unidentified receptor (1) and may be internalized (2). Inflammasome adaptor proteins (likely ASC/CARD) associate with this endosome (3) and recruit procaspase-1 (Pro-casp-1), which is auto-catalytically activated (4). Active caspase-1 can cleave pro-IL-1 into its mature form (5). Mature IL-1β is secreted (6), such that it can then bind to the IL-1 receptor, IL-1R (7). Signaling through MyD88, IL-1R activates NFκB (8), as does downstream signaling from the inflammasome (9). NFκB mediates transcription of inflammatory genes (10), resulting in a redistribution of tight junction and adherens junction proteins and a compromised blood-brain barrier (11), as well as in an increase in levels of surface adhesion molecules (12).

The data suggest a model for HRPII action on endothelial cells (Fig. 6). HRPII accumulates in the bloodstream and binds to vascular endothelium via an unknown receptor. Downstream signaling allows recruitment of inflammasome components, which activate caspase-1, resulting in cleavage of substrates, including pro-IL-1β, yielding mature IL-1β. Active IL-1β is secreted, at which point it can bind to cell surface receptor IL-1R. IL-1R ligation transmits a MyD88-dependent signal that activates the transcription factor NFκB. NFκB translocates to the nucleus and induces transcription of many genes, including cytoskeletal components, which can redistribute tight junction and adherens junction proteins (47) and can alter surface expression of adhesion receptors such as ICAM-1 and VCAM-1. Although there have been reports of IL-1-mediated tight junction rearrangement via an NFκB-independent pathway involving ARNO/Arf6 signaling (48), HRPII activity has a different time course and requires NFκB signaling to impact barrier integrity.

Infection of mice with the rodent malaria parasite strain *P. berghei* ANKA serves as a small-animal model for cerebral malaria. The pathology present in experimental cerebral malaria (ECM) is similar to that in human cerebral malaria (CM), with notable exceptions being fewer sequestered infected RBCs and a more robust infiltration of leukocytes (5, 49). The biological basis of these differences is controversial and poorly defined (5, 50, 54). Aspects of HRPII action may explain some differences between the pathophysiologies seen in human CM and ECM, since *P. berghei* lacks an HRPII gene. For example, tight junction protein relocalization is not observed in ECM, suggesting that vascular leakage in the rodent model occurs via a mechanism other than that observed in humans (55, 56). HRPII-mediated barrier compromise depends on caspase-1 activation of IL-1β, and IL-1β levels in the cerebrospinal fluid of patients correlate with disease severity (57) whereas IL-1β has been shown to be dispensable for the experimental CM model (58).

Blocking HRPII action could be a prophylactic or therapeutic strategy. Results of attempts in the 1980s to use HRPII as an immunogen in Aotus monkey vaccination trials were encouraging, but results of follow-up studies were equivocal (59–61). New studies informed by the proposed role in cerebral malaria could lead to development of a vaccine that prevents CM. HRPII-induced damage might be minimized by targeting upstream components of the pathway. Drugs targeting caspase-1 and IL-1β are already in clinical use and could be considered for the treatment of cerebral malaria.

Since causing CM is not likely to benefit the parasite, the question of why *P. falciparum* has evolved and maintained the HRPII gene is germane. One possible advantage for the parasite is that triggering an inflammatory pathway leads to increased expression of cytoadherence molecules on the endothelial surface. Cytoadherence allows the parasite to avoid clearance in the spleen and to reside in a low-oxygen, high-carbon-dioxide environment. Parasite sequestration in the cerebral vasculature is the hallmark feature of human CM and one that is largely absent in the murine experimental CM model. Other cytokines such as tumor necrosis factor alpha (TNF-α) also upregulate adhesion molecules that are receptors for *P. falciparum*-infected erythrocytes (62, 63).

Several new questions arise from this work. What does HRPII bind, to initiate the inflammatory cascade? Is HRPII synergistic with other *P. falciparum* virulence factors such as glycosylphosphatidylinositol (64)? Does HRPII affect endothelium in other vascular beds in a similar manner? Our data suggest that HRPII may contribute to malaria pathogenesis by modulating the BBB; further mechanistic insight is needed to develop novel HRPII-dependent pharmacological or vaccine-based strategies for disease control.

## MATERIALS AND METHODS

### Reagents.

Bovine serum albumin (BSA) of reagent grade was from Sigma. Lipopolysaccharide (LPS) from *Escherichia coli* O111:B4 was purchased from List Biological Laboratories, Inc.

### Antibodies.

Mouse anti-HRPII (2G12), a generous gift from Diane Taylor (University of Hawaii), was used at 1:100, goat anti-claudin-5 (from Santa Cruz; sc-17667) at 1:100, and VE-cadherin (Santa Cruz; sc-52751) at 1:100. Five micrograms of anti-HRPII was used for neutralization and was from Thermo Scientific (MA1-27094), and the isotype control (269) was generated as described previously (65). Rabbit anti-IL-1β (Rockland Immunochemical; 209-401-301) was used for neutralization. Rabbit anti-GAPDH (anti-glyceraldehyde-3-phosphate dehydrogenase) (Abcam; ab37168) was used at 1:1,000. Mouse anti-ICAM-1, anti-VCAM-1, and anti-E-selectin (BD Biosciences; 555510, 555645, and 555648) were used at 1:500, 1:100, and 1:500 dilutions, respectively. Armenian hamster anti-IL-1β (Leinco) was used for *in vivo* neutralization at 300 µg/mouse.

### Inhibitors.

Triptolide (InvivoGen) was used at a final concentration of 100 nM. Celastrol (InvivoGen) was used at a final concentration of 8.8 µM. IL-1Ra (Sigma) was used at a final concentration of 500 ng/ml. Caspase-1 inhibitor (Sigma, SML0429) was used at a final concentration of 80 µM.

### HRPII purification.

The coding sequence for the mature form of HRPII was cloned into the pet-15b vector (Novagen) without a tag, expressed, and purified from *E. coli* lysate using nickel resin as previously described (17). Protein was exchanged into 20 mM Tris–500 mM NaCl–50 mM imidazole and loaded on a 5-ml nickel fast protein liquid chromatography (FPLC) column (GE Healthcare). After washing with 60 column volumes of 20 mM Tris–10 mM NaCl–0.1% Triton X-114 to remove residual LPS was performed, the column was washed with 20 column volumes of loading buffer and eluted with loading buffer with 1 M imidazole. All preparations of HRPII were tested for residual LPS using a LAL endotoxin test (Charles River Laboratories; R1708K). Antithrombin-inhibitory activity was measured using a Factor Xa assay (17). Protein concentration was determined by BCA assay (Fisher). Fully active, pure preparations of HRPII protein were used in all experiments.

### *P. falciparum* transfection.

HRPII was PCR amplified from 3D7 parasite genomic DNA and cloned into TOPO vector (Life Technologies). HRPII was inserted upstream of green fluorescent protein (GFP) in the tEOE vector under control of the Hsp86 promoter. This vector is a modified form of tyEOE vector with the selectable marker human dihydrofolate reductase replacing the yeast dihydroorotate dehydrogenase selection cassette (66). Dd2 transfections were performed as previously reported, and parasites were selected with 10 nM WR99210 and cloned (67). Clones were screened by PCR.

### *In vitro* BBB cultures and TEER recordings.

*In vitro* BBB endothelial cell cultures were prepared as previously described (30). Briefly, 10^5^ hCMEC/D3 cells (68) were cultured. Components for assessment (recombinant or native protein, chemical inhibitors, and parasitized erythrocytes) were added to the apical chamber immediately after determining baseline values for each well. Resistance recordings were measured via chopstick electrode with an EVOM voltmeter (World Precision Instruments). Resistance values are expressed in ohms per square centimeter.

### ShRNA knockdown and TEER.

hCMEC/D3 cells (10^5^) were cultured on the apical side of a 0.9-cm^2^ fibronectin-coated polyethylene terephthalate filter insertion with 3.0-µm porosity (BD Falcon). At 24 h later, cells were transfected with 500 ng of shRNA and with Lipofectamine 3000 at a 1.5:1 ratio of Lipofectamine to DNA. Cells were then incubated for 36 h. HRPII was then added, and TEER measurements were recorded over 24 h. shRNAs for each gene were purchased from Origene as follows: for the Myd88 gene, TG311320; for the NFκB gene, TR318700; for the caspase-1 gene, TG305640; for the TLR9 gene, TR301076; for the TLR5 gene, TR308792; and for the TLR2 gene, TR320553. Of the four shRNAs to each gene received from the vendor, 2 to 3 of each were used based on silencing efficiency in pilot studies. M5 for the MyD88 gene was from InvivoGen (ksirna42-hmyd88 [M5 in this study]). Silencing efficiency for all assays was determined by qRT-PCR.

### Quantitative RT-PCR.

Total RNA was isolated from treated or untreated cultured hCMEC/d3 cells using an RNeasy kit (Qiagen). To remove DNA, samples were treated with RNase-free DNase (Qiagen). mRNA was quantified from total RNA by qRT-PCR as previously described (69). Primetime quantitative PCR (qPCR) primers and probes were purchased from IDT. GAPDH mRNA expression levels also were determined, and normalization was performed using the threshold cycle (*C_T_*) method as previously described (69).

### Immunocytochemical analysis.

Immunocytochemical analysis was performed on hCMEC/D3 cells posttreatment with recombinant HRPII or controls after a 10-min fixation in ice-cold methanol, followed by blocking in 3% BSA at room temperature. Cells then were incubated with primary antibodies in blocking buffer, washed three times in phosphate-buffered saline (PBS), and then incubated in secondary Alexa Fluor-conjugated antibodies in blocking buffer at room temperature. Slides were washed and then, in some cases, stained with To-Pro at a 1:500 dilution. Sections were sealed with ProLong Gold antifade, and then images were acquired by confocal microscopy (Carl Zeiss, USA).

Terminal deoxynucleotidyltransferase-mediated dUTP-biotin nick end labeling (TUNEL) using a TUNEL assay kit (Roche; 12156792910) was performed on paraformaldehyde-fixed hCMEC/D3 cells that had been treated with 25 µg recombinant HRPII, control protein, or 3 µg LPS for 6 to 24 h.

### HRPII internalization.

hCMEC/D3 cells were incubated with 1 µg HRPII–1 ml medium for 5 min at 0°C or 37°C. Cultures were washed and incubated for another 25 min at the same temperature in medium lacking HRPII. Control incubations lacked HRPII. Cells were fixed as before in 100% methanol and stained for HRPII using the 2G12 monoclonal Ab (1:100 dilution) or secondary Ms-488 (1:1,000). Slides were sealed with ProLong Gold antifade, and images were acquired.

## SUPPLEMENTAL MATERIAL

Figure S1  Dd2 transgenic parasite clones C5 and D3 have been successfully transfected with the gene encoding HRPII-GFP and produce protein. (A) PCR of the HRPII gene from wild-type Dd2 parasites (lanes 1 and 4) as well as the transgenic clone 1 (lanes 2 and 5) and clone 2 (lanes 3 and 6), amplifying for HRPII (lanes 1 to 3) and HRPII-GFP (lanes 4 to 6). (B) Western blots of parasite extracts from wild-type Dd2 (lane1), 3D7 (lane 2), clone C5 (lane 3), and clone D3 (lane 4) using anti-HRPII (clone 2G12; 1:10,000). The yellow box highlights the band for HRPII-GFP, and the white box highlights native untagged HRPII. The two transgenic clones gave similar results in the TEER assay. Download Figure S1, TIF file, 14.5 MB

Figure S2  HRPII-mediated BBB compromise requires protein synthesis and cannot be mimicked with high histidine content. (A) TEER measurements for *in vitro* BBB models treated with IFN-γ (100 ng/ml) or HRPII (50 µg) or left untreated (solid lines). Barriers were also pretreated with cycloheximide (1 mg/ml) for 30 min prior to addition of IFN-γ (100 ng/ml) or HRPII (50 µg) (dashed lines). (B) TEER measurements for *in vitro* BBB models treated with HRPII (10 µg), IFN-γ (100 ng/ml), and equimolar poly-l-histidine, l-histidine, HHPP-3 (HHAHHAADAHHAHHAADA), and HHPP-4 (HHAADHHAAD) at 24 h. Download Figure S2, TIF file, 7.4 MB

Figure S3  Degree of gene silencing by various shRNAs. shRNAs to TLR2 (2-1 and 2-2), TLR5 (5-3 and 5-4), TLR9 (9-3 and 9-4), NFkB (N1 and N3), to Myd88 (M1 and M3 and M5), to caspase-1 (C1 and C2) were used. hCMEC/D3 cells were incubated with shRNAs as described for Fig. 3 (see also Fig. S4). mRNA levels were quantified by qRT-PCR. Data shown are from triplicate determinations. Values are normalized for the percentages of cells transfected, as determined from visualization of GFP-expressing shRNA by flow cytometry. Data are means of results from 3 replicates (TLR5), 4 replicates (TLR9, NFkB, Myd88, caspase-1), or 5 replicates (TLR2) ± SEM determined over three independent experiments. Download Figure S3, TIF file, 5 MB

Figure S4  HRPII-mediated BBB compromise does not require TLR2, TLR5, or TLR9. Data represent results of TEER measurements for *in vitro* BBB models transfected with scrambled control (Scrb) or shRNAs to TLR2 (2), TLR5 (70), and TLR9 (70), alone or with HRPII (+ H, 10 µg). Data are means of results from 5 to 7 replicates ± SEM determined over three independent experiments. Download Figure S4, TIF file, 3.9 MB

Figure S5  HRPII binds to and is internalized by hCMEC/D3 endothelial cells. Cells were incubated with 1 µg HRPII in 1 ml of medium for 5 min at 0° or 37°C. Control incubations lacked HRPII. Cultures were washed and incubated for another 25 min at the same temperature in medium lacking HRPII. Cells were fixed, stained with anti-HRPII antibody, and processed for immunofluorescence. Top panels, HRPII added; bottom panels, no HRPII controls. The 37°C incubation showed a vesicular pattern, while the 0°C incubation gave a diffuse surface pattern. Images are representative of results from four replicates determined over two independent experiments. Download Figure S5, TIF file, 16.6 MB
