# Supplementary figures and images for: Plasmodium falciparum Histidine-Rich Protein II Compromises Brain Endothelial Barriers and May Promote Cerebral Malaria Pathogenesis
Source: mBio. 2016 Jun 7;7(3):e00617-16. doi: 10.1128/mBio.00617-16 (PMC4959673; doi:10.1128/mBio.00617-16)

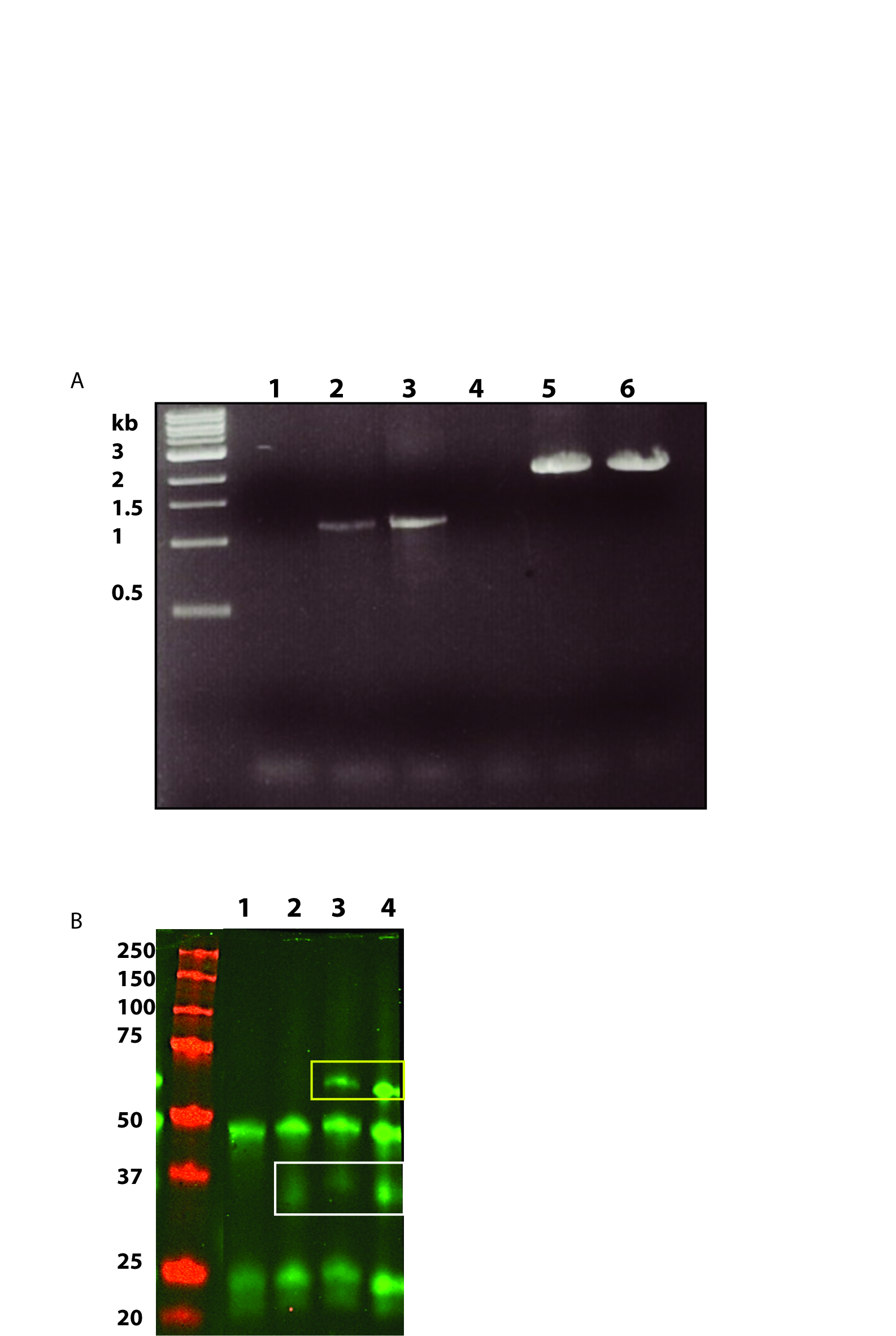

Supplement: Figure S1 — Dd2 transgenic parasite clones C5 and D3 have been successfully transfected with the gene encoding HRPII-GFP and produce protein. (A) PCR of the HRPII gene from wild-type Dd2 parasites (lanes 1 and 4) as well as the transgenic clone 1 (lanes 2 and 5) and clone 2 (lanes 3 and 6), amplifying for HRPII (lanes 1 to 3) and HRPII-GFP (lanes 4 to 6). (B) Western blots of parasite extracts from wild-type Dd2 (lane1), 3D7 (lane 2), clone C5 (lane 3), and clone D3 (lane 4) using anti-HRPII (clone 2G12; 1:10,000). The yellow box highlights the band for HRPII-GFP, and the white box highlights native untagged HRPII. The two transgenic clones gave similar results in the TEER assay. Download [file mbo003162855sf1.tif]

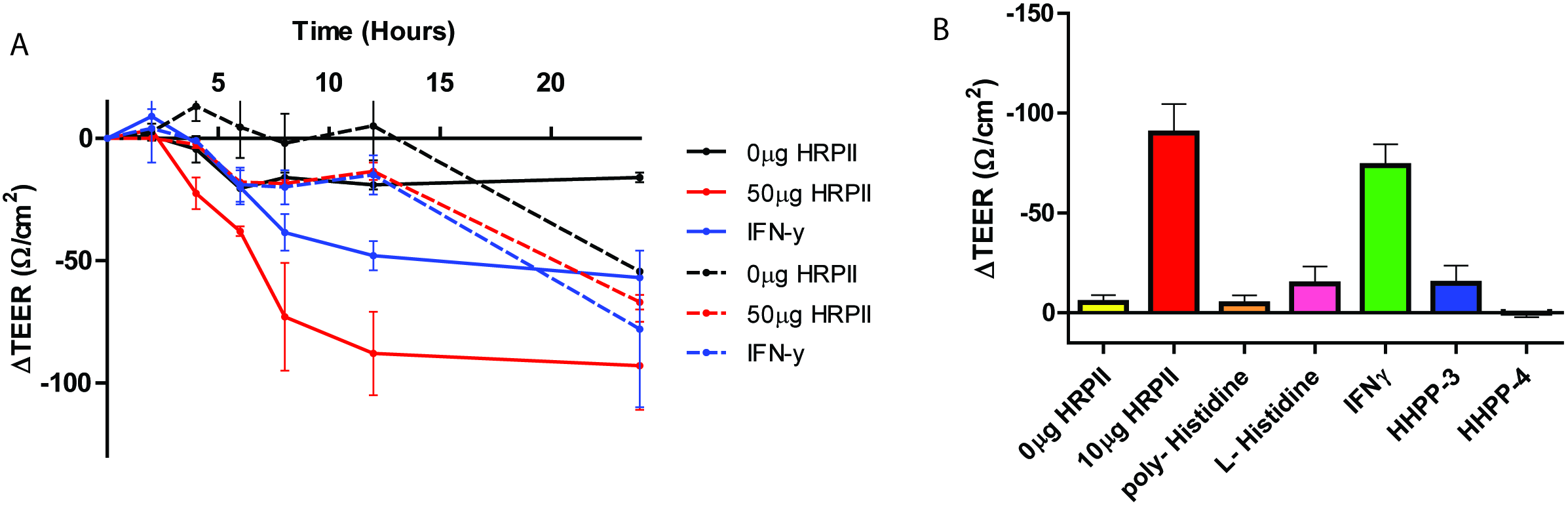

Supplement: Figure S2 — HRPII-mediated BBB compromise requires protein synthesis and cannot be mimicked with high histidine content. (A) TEER measurements for in vitro BBB models treated with IFN-γ (100 ng/ml) or HRPII (50 µg) or left untreated (solid lines). Barriers were also pretreated with cycloheximide (1 mg/ml) for 30 min prior to addition of IFN-γ (100 ng/ml) or HRPII (50 µg) (dashed lines). (B) TEER measurements for in vitro BBB models treated with HRPII (10 µg), IFN-γ (100 ng/ml), and equimolar poly-l-histidine, l-histidine, HHPP-3 (HHAHHAADAHHAHHAADA), and HHPP-4 (HHAADHHAAD) at 24 h. Download [file mbo003162855sf2.tif]

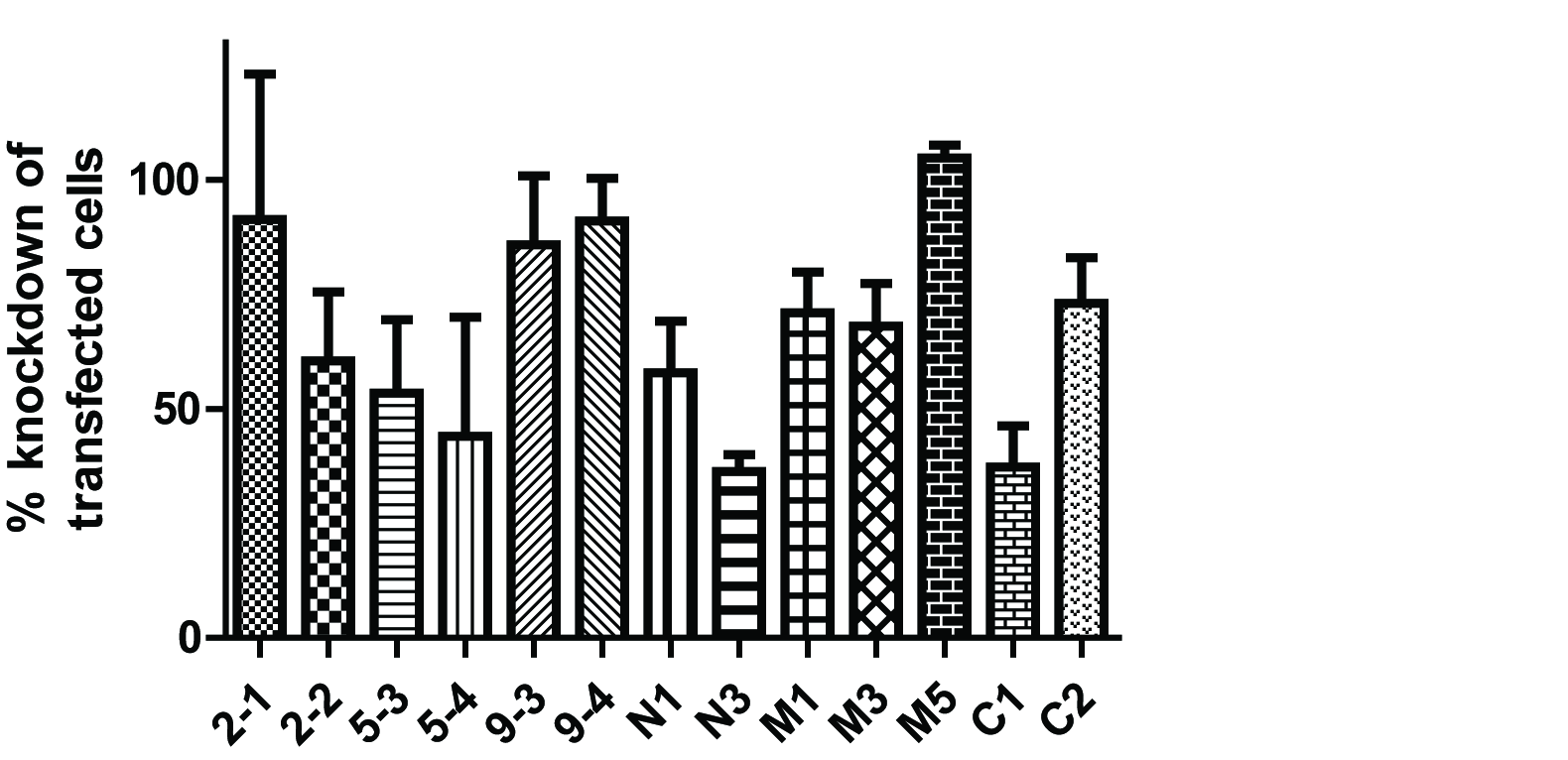

Supplement: Figure S3 — Degree of gene silencing by various shRNAs. shRNAs to TLR2 (2-1 and 2-2), TLR5 (5-3 and 5-4), TLR9 (9-3 and 9-4), NFkB (N1 and N3), to Myd88 (M1 and M3 and M5), to caspase-1 (C1 and C2) were used. hCMEC/D3 cells were incubated with shRNAs as described for Fig. 3 (see also Fig. S4). mRNA levels were quantified by qRT-PCR. Data shown are from triplicate determinations. Values are normalized for the percentages of cells transfected, as determined from visualization of GFP-expressing shRNA by flow cytometry. Data are means of results from 3 replicates (TLR5), 4 replicates (TLR9, NFkB, Myd88, caspase-1), or 5 replicates (TLR2) ± SEM determined over three independent experiments. Download [file mbo003162855sf3.tif]

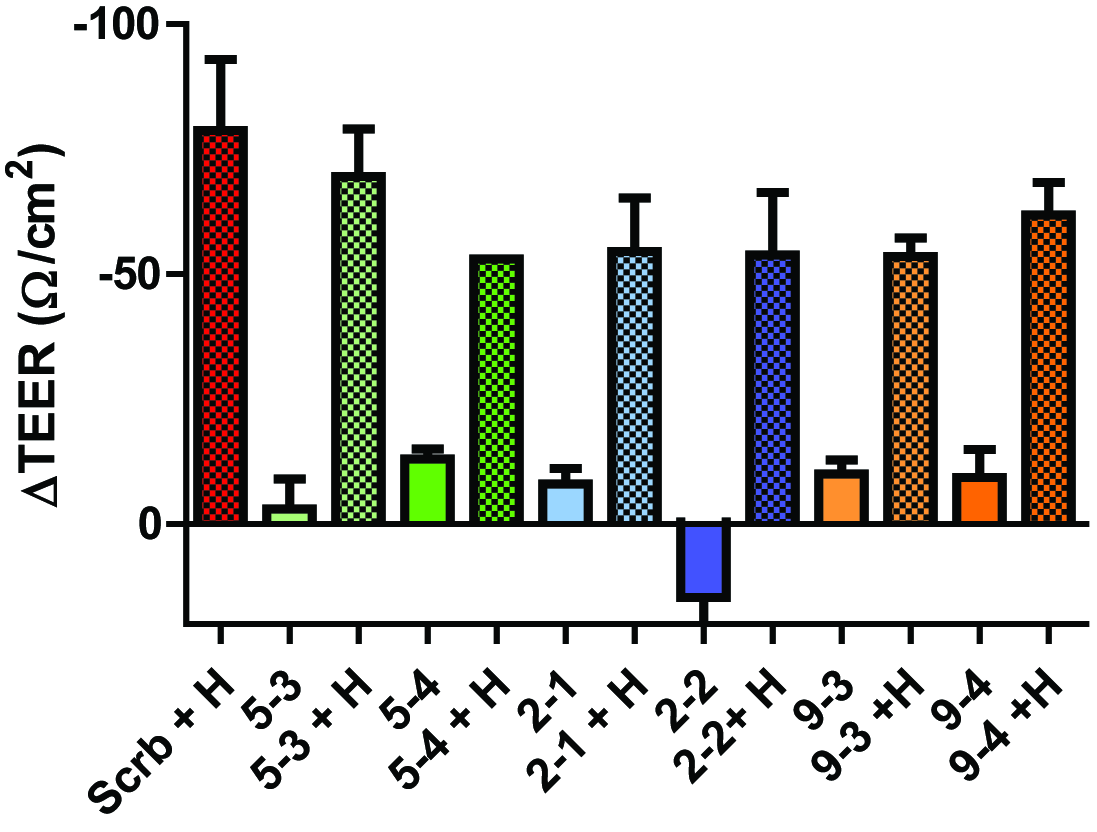

Supplement: Figure S4 — HRPII-mediated BBB compromise does not require TLR2, TLR5, or TLR9. Data represent results of TEER measurements for in vitro BBB models transfected with scrambled control (Scrb) or shRNAs to TLR2 (2), TLR5 (70), and TLR9 (70), alone or with HRPII (+ H, 10 µg). Data are means of results from 5 to 7 replicates ± SEM determined over three independent experiments. Download [file mbo003162855sf4.tif]

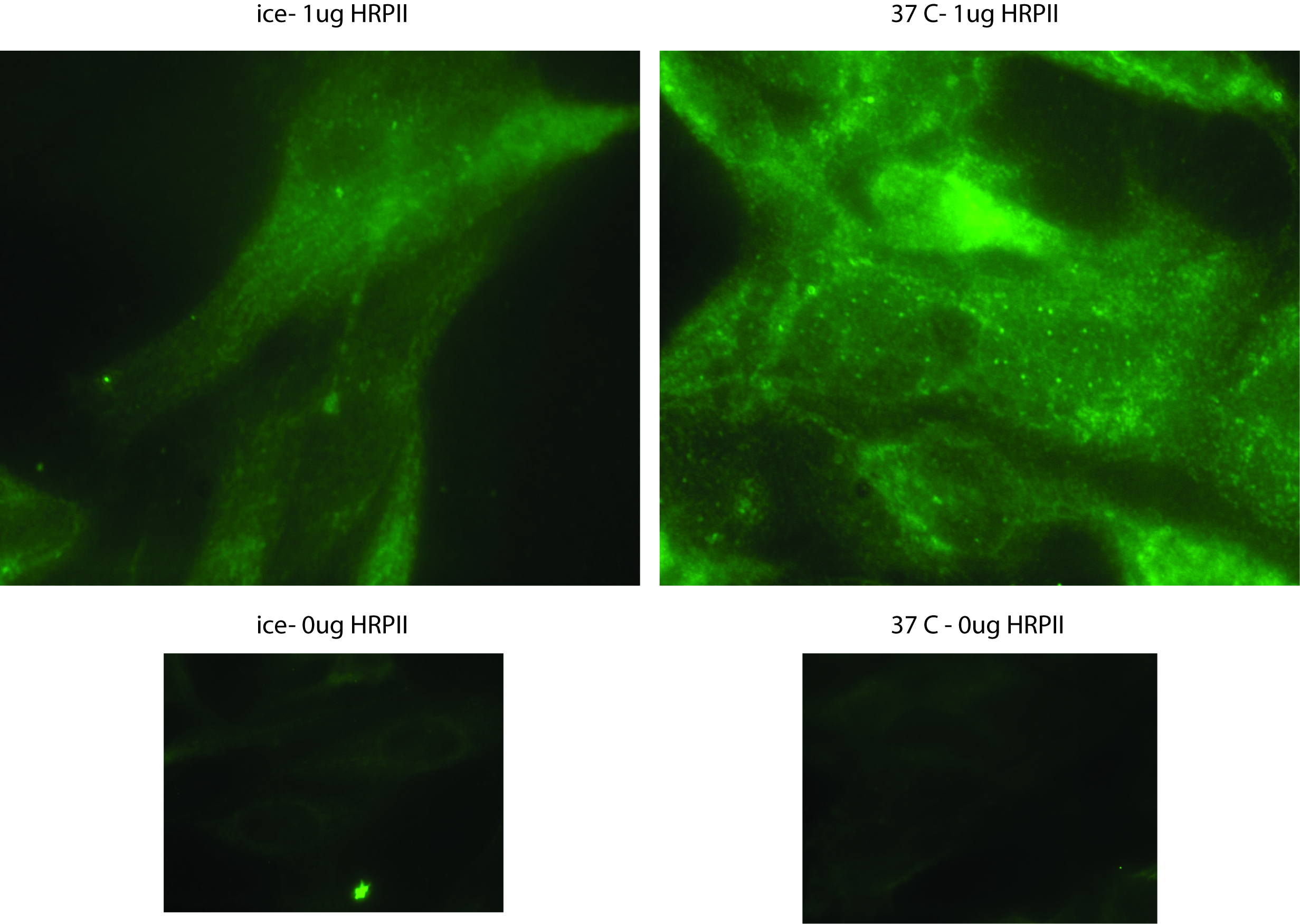

Supplement: Figure S5 — HRPII binds to and is internalized by hCMEC/D3 endothelial cells. Cells were incubated with 1 µg HRPII in 1 ml of medium for 5 min at 0° or 37°C. Control incubations lacked HRPII. Cultures were washed and incubated for another 25 min at the same temperature in medium lacking HRPII. Cells were fixed, stained with anti-HRPII antibody, and processed for immunofluorescence. Top panels, HRPII added; bottom panels, no HRPII controls. The 37°C incubation showed a vesicular pattern, while the 0°C incubation gave a diffuse surface pattern. Images are representative of results from four replicates determined over two independent experiments. Download [file mbo003162855sf5.tif]
